# Use of Machine Learning in the Analysis of Indoor ELF MF Exposure in Children

**DOI:** 10.3390/ijerph16071230

**Published:** 2019-04-06

**Authors:** Gabriella Tognola, Marta Bonato, Emma Chiaramello, Serena Fiocchi, Isabelle Magne, Martine Souques, Marta Parazzini, Paolo Ravazzani

**Affiliations:** 1 CNR IEIIT—Consiglio Nazionale delle Ricerche, Istituto di Elettronica e di Ingegneria dell’Informazione e delle Telecomunicazioni, 20133 Milan, Italy; marta.bonato@ieiit.cnr.it (M.B.); emma.chiaramello@ieiit.cnr.it (E.C.); serena.fiocchi@ieiit.cnr.it (S.F.); marta.parazzini@ieiit.cnr.it (M.P.); paolo.ravazzani@ieiit.cnr.it (P.R.); 2 Dipartimento di Elettronica, Informazione e Bioingegneria DEIB, Politecnico di Milano, 20133 Milan, Italy; 3 EDF Electricite de France, 92300 Levallois-Perret, France; isabelle.magne@edf.fr (I.M.); martine.souques@edf.fr (M.S.)

**Keywords:** children, ELF MF, magnetic field, indoor exposure, cluster analysis, Machine Learning

## Abstract

Characterization of children exposure to extremely low frequency (ELF) magnetic fields is an important issue because of the possible correlation of leukemia onset with ELF exposure. Cluster analysis—a Machine Learning approach—was applied on personal exposure measurements from 977 children in France to characterize real-life ELF exposure scenarios. Electric networks near the child’s home or school were considered as environmental factors characterizing the exposure scenarios. The following clusters were identified: children with the highest exposure living 120–200 m from 225 kV/400 kV overhead lines; children with mid-to-high exposure living 70–100 m from 63 kV/150 kV overhead lines; children with mid-to-low exposure living 40 m from 400 V/20 kV substations and underground networks; children with the lowest exposure and the lowest number of electric networks in the vicinity. 63–225 kV underground networks within 20 m and 400 V/20 kV overhead lines within 40 m played a marginal role in differentiating exposure clusters. Cluster analysis is a viable approach to discovering variables best characterizing the exposure scenarios and thus it might be potentially useful to better tailor epidemiological studies. The present study did not assess the impact of indoor sources of exposure, which should be addressed in a further study.

## 1. Introduction

Interest in the analysis of children exposure to extremely low frequency magnetic fields (ELF-MF, 40–800 Hz) was raised with the first epidemiological study where exposure to ELF was found to be a possible health risk factor for childhood leukemia [1]. Although leukemia is the most common type of cancer in children younger than 15 years, its etiology is still unknown. Previous studies evidenced that daily average exposures >0.4 μT increased the risk of childhood leukemia’s onset, without any causal relationship [2,3,4]. To further investigate their possible relationship with children leukemia, several studies were conducted to measure personal exposure to ELF in children in Europe [5,6,7,8,9], United States [10,11,12,13,14,15], and Asia [16,17,18].

The aim of the present study was to gain deeper insights on the variables (such as, sources of ELF in the environment) that characterize real-life exposure scenarios and better characterize ELF exposure in children. In particular, we focused on the characterization of electric networks in the proximity of a child’s home or school as environmental factors that potentially could influence indoor exposure.

We used cluster analysis, a non-parametric and unsupervised Machine Learning (ML) approach. In contrast to parametric approaches, such as correlation analysis or multivariate analysis, cluster analysis is applied in exploratory data mining to understand possible relationships within the data without assuming any linear or non-linear parametric model to explain the observed data. The application of ML approaches to problems related to electromagnetic field (EMF) exposure is at the very beginning; to the best of our knowledge, sporadic examples of applications can be found in the prediction of radiofrequency (RF) radiation effect on plants [19] or the prediction of wireless local-area network (LAN) EMF in the indoor environment [20].

This is the first time that ML has been used for the characterization of ELF exposure scenarios in children. Cluster analysis was applied to personal exposure measurements recorded in the EXPERS study [5,6] from children living in France to identify clusters of children with similar exposure levels and similar exposure scenarios (i.e., similar electric network configuration in the proximity of the home or school). The aim of the paper was neither to demonstrate the influence of ELF exposure on children Leukemia, nor to present a novel measurement study of personal ELF exposure. The aim was to further analyze data coming from an already done measurement campaign—the EXPERS study—with a novel approach in order to discover similarities in the exposure scenarios. In particular, the focus was to understand (i) what were the main characteristics of the clusters of ELF exposure with respect to the type of electric networks in the vicinity of the measurement location (i.e., a child’s home and school), (ii) what were the main differences between the clusters, and (iii) which environmental factors better discriminated the clusters. The paper is organized as follows: Section 2 “Materials and Methods” reports the details of the source of the analyzed data (Section 2.1 Data Source) and the description of the method used to perform cluster analysis and its validation (Section 2.2 Data Analysis); Section 3 “Results” reports some statistics on the analyzed dataset (Section 3.1 Descriptive Results), the results of the procedure used to determine the optimal number of clusters and the evaluation of the cluster solution (Section 3.2 Choice of the Optimal Number of Clusters and Evaluation of Clustering Performance), and the detailed description of the characteristics of the clusters we found (Section 3.3 Results of Clustering Analysis); Section 4 “Discussion” reports the discussion of the results, current study limitations and future directions; the last section, Section 5 “Conclusions”, summarizes the main findings of the study.

## 2. Materials and Methods

### 2.1. Data Source

A database of personal exposure measurements to ELF MF in children was analyzed. The data came from the EXPERS study [5,6] and consisted of exposure measurements recorded from children aged 0–14 years located in France. The database contained the recordings from 977 children located in rural or urban places (948 children) and in Paris (29 children) (for more details about the areas under test, see [6]). Measurements were performed during cold seasons (February–April 2007, October 2007–April 2008, and October 2008–January 2009). An EMDEX II (Enertech, Campbell, CA, USA (https://www.enertech.net/html/EMDEXII.html)) personal dosimeter was used to measure broadband (40–800 Hz) and harmonic (100–800 Hz) MF amplitudes with a sensitivity of 0.01–300 µT. Measurements were recorded with a sampling rate of 3 s over 24 h for one day. For each child, the database contained the number of power lines and substations close to the measurement site, i.e., the child’s home or school, as identified by the French grid operators (RTE for high voltage and Enedis for mid and low voltage) from the Lambert II coordinates of home and school addresses. The following 10 types of the electric networks were considered: 400 kV overhead lines within 200 m from home or school (OVHD_ultra-high), 225 kV overhead lines within 120 m (OVHD_extra-high), 150 kV or 90 or 63 kV overhead lines within 100 m (for 150 kV lines) or 70 m (for 63 and 90 kV lines) (OVHD_high), 20 kV overhead lines within 40 m (OVHD_mid), 400 V overhead lines within 40 m (OVHD_low), 225 kV underground cables within 20 m (UND_extra-high), 63 to 150 kV underground cables within 20 m (UND_high), 20 kV underground cables within 40 m (UND_mid), 400 V underground cables within 40 m (UND_low), and 20 kV/400 V substations inside a building within 40 m (Substation). Also, the database contained a timetable reporting time, duration, and location (e.g., at home, at school, indoors, outdoors, etc.) of child activities during the 24 h of exposure measurement.

### 2.2. Data Analysis

Using the information available in the daily activity tables, the geometric mean (GM) of the 50 Hz component of B field was computed for exposure measurements that were recorded indoors when the child was at home and, where available, at school. As in previous reference studies on ELF MF exposure [21], we decided to consider GM as the metric to represent B field values to facilitate the comparison of our results with similar studies. We also decided to analyze only exposures recorded during the day and not during the night to exclude any confounding effect due to the presence of the alarm clock in night recordings, as evidenced in [6]. As written above, in this study, we decided to analyze as variables the B field measured indoor and the type of electric networks in the vicinity of the measurement site; at the moment, we did not analyze indoor sources of exposure. A total of 1793 measurements were available for the analysis, including 977 recordings made at home and 816 at school.

Cluster analysis was performed on a matrix of 1793 × 11 real data, each representing a child exposure (row) and its characterization (column) in terms of B field and number of power lines and substations in close proximity to home or school for each of the 10 types of the electric networks described in the section above.

Data were analyzed with *K*-means clustering (Matlab (ver. R2018a), MatWorks Inc., Natick MA, USA) [22]. The goal of *K*-means clustering is to partition the data into *K* clusters so that the pairwise dissimilarities between data of the same cluster are smaller than those in the other clusters. In *K*-means clustering, dissimilarity $d(x_{i}$, $\;x_{i\prime })$ between two data vectors $x_{i}$ and $x_{i\prime }$ (i,i′=1,⋯,N) of *P* components is measured with the squared Euclidean distance:

(1) $$\;d(x_{i},x_{i\prime })=\sum_{j=1}^{P}{\left(x_{ij}-x_{i\prime j}\right)}^{2},$$

where $x_{ij}$ and $x_{i\prime j}$ are the *j*th components (j  =1,⋯,P) of vectors $x_{i}$ and $x_{i\prime }$.

In *K*-means clustering, the user has to specify the number of partitions *K* < *N* into which the data points have to be clustered; then, the *K*-means algorithm assigns each data point to one and only one of the *K* clusters by minimizing the so-called “within cluster” point scatter *W*(*C*):

(2) $$W\left(C\right)=\frac{1}{2}\sum_{k=1}^{K}\sum_{i,\;i^{\prime }\in Sk}d\left(x_{i},x_{i^{\prime }}\right),$$

where *Sk* is the set of observations in the *k*th cluster (k=1,⋯,K). Minimization of *W*(*C*) is obtained by an iterative procedure that at each step searches among the possible assignments of the *N* data points to the *K* clusters that one that improves (i.e., minimizes) *W*(*C*) from its value obtained at the previous step. The iteration is stopped when the new assignment solution is unable to improve *W*(*C*) further. In *K*-means clustering, it is possible to demonstrate [22] that minimization of (2) is equivalent to minimizing the average distance of the data points of the same cluster from the cluster centroid ${\bar{x}}_{k}=\left({\bar{x}}_{1k},\cdots ,{\bar{x}}_{Pk}\right)$, which is the mean vector of the data assigned to the same cluster. As such, each cluster centroid can be regarded as the “representative” of the characteristics of the pool of data assigned to the cluster. Centroid initialization was obtained from the algorithm published by [23] (as implemented in Matlab), which uses heuristics to find centroid seeds and improves the running times of the *K*-means clustering algorithm. To be sure that the partitioning solution was stable, we repeated the clustering using new initial cluster centroid positions by choosing from 30 possibly different sets of seeds, according to the algorithm by [23].

The average silhouette score was used to determine *K* (i.e., the optimal number of clusters) and the appropriateness of the cluster solution (i.e., it was used for the validation of the cluster solution). The silhouette score $S_{i}$ for a data point $x_{i}$ is a measure of the similarity between $x_{i}$ and the other data of the same cluster, when compared to data in other clusters [24]. It is computed as:

(3) $$S_{i}=\frac{b_{i}-a_{i}}{\max\left(a_{i},b_{i}\right)},$$

where $a_{i}$ is the average distance of $x_{i}$ to the other points in the same cluster, $b_{i}$ is the minimum average distance of $x_{i}$ to all the other points in different clusters, minimized over the clusters, and $-1\leq S_{i}\leq 1$. A high silhouette value close to 1 indicates that $x_{i}$ is far away from neighboring clusters and well-matched to its own cluster, that is, $x_{i}$ has been assigned to the proper cluster. A value close to −1 indicates that $x_{i}$ has been assigned to the wrong cluster. The average of $S_{i}$ across all data points (*i*  =1,⋯,N) is a measure of the overall appropriateness of the clustering solution over the entire dataset. The average silhouette score can be used to determine the optimal number of clusters *K*: by computing the average silhouette score as a function of *K*, it is possible to determine the best value of *K* as the value for which the average silhouette reaches a maximum. The value of the average silhouette score is also used as a metric to assess the robustness of the clustering solution, with values greater than 0.5 indicating a good quality in the partitioning of the data.

## 3. Results

### 3.1. Descriptive Results

The average value of B field (GM) across all 1793 recordings was 0.02 μT (1st quartile Q1: 0.006 μT; 3rd quartile Q3: 0.042 μT), and the maximum was 1.09 μT; one should note that B field was >0.4 μT only in 5 (five) recordings out of 1793.

Table 1 shows the distribution of the different types of electric networks in the analyzed dataset of indoor measurements. It is possible to see that more than two thirds of the measurements (66.8%) in the dataset came from children living or going to schools near underground networks of low voltage, while nearly half of them were from children living or going to school near underground networks of mid voltage (45.7%) or overhead lines of low voltage (43.8%), and fewer than 15% (13.7%) of the measurements were from children living or going to school near a substation. A small number of measurements (below 4%) were from children living or going to school near underground networks of high or extra-high voltage or overhead lines of mid to ultra-high voltage. A total of 201 out of 1793 (11.2%) measurements came from sites that apparently were far from any of the electric networks considered in the present study; in other words, none of the electric networks mentioned above was found near the measurement site of these last data. This group of data may consist of measurements from places served by local distributors of electricity rather than by RTE and Enedis, representing 5% of the distribution of electricity in France. In addition, this group included measurements made in rural areas where the precision of geolocalization might have been less than in urban areas, and thus might have influenced the localization of the electric grid near the measurement site, especially if the precision was larger than 40 m.

Table 1 also shows the maximum and mean number of power lines and substations found in the proximity of the measurement site. The maximum number of underground cables of low voltage close to the measurement site was 59, followed by 27 underground cables of mid voltage and 16 overhead lines of low voltage; the number of underground cables of high or extra-high voltage or overhead lines of mid to ultra-high voltage or substations close to the measurement site was at maximum five.

The analysis of the dataset also revealed that a significant percentage of measurements came from children who live or go to schools near two different types of electric networks at the same time. Data in Table 2 show that children who lived near underground networks of mid voltage also had underground networks of low voltage in the vicinity (38.8% of the total number of measurements); as expected by grid construction, those near a substation were found to have underground networks of low (12.7% of the measurements) or mid voltage (13.2% of the measurements) and overhead lines of low voltage (4.9% of the measurements) in the vicinity; those near an overhead line of low voltage also had underground networks of low (24%) or mid voltage (19.6%) in the vicinity; children living or going to schools near overhead lines of mid to ultra-high voltage were found to have underground networks of low and mid voltage (up to 1.2% of total number of measurements) and overhead lines of low voltage (up to 2.3%) in the vicinity. None of the children near overhead lines of mid to ultra-high voltage lived or went to schools that are also in the vicinity of underground networks of high and extra-high voltage. This is another finding which is due to construction on the 63 kV–400 kV grid; as a matter of fact, it is very rare, except for sites near high voltage substations, to live near underground and overhead lines of 63 kV–400 kV at the same time. Overhead lines of this type are mainly found in the country side, whereas underground lines are found in big cities.

**Figure 1:**
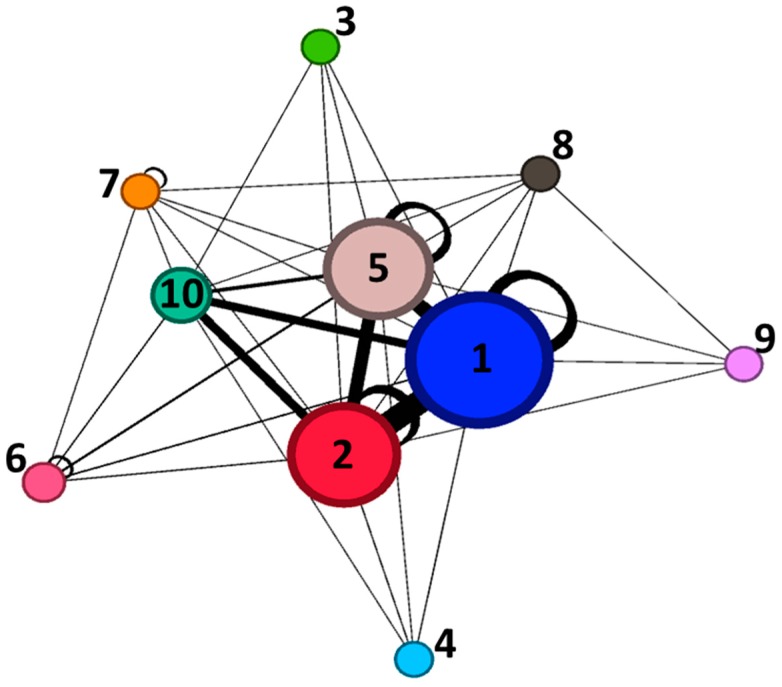
Pictorial representation of the distribution of electric networks in the analyzed dataset and their interconnections. Nodes (circles) represent the electric networks. Node size is proportional to the number of children in the dataset living or going to a school near that particular electric network. A link (straight line) between electric networks “A” and “B” means that there are some children in the dataset whose home or school is near both “A” and “B” networks at the same time. Loop links represent children that are near to only one single type of electric networks. Link thickness is proportional to the number of children for which the link is valid. Numbers in or next to the nodes are the type of electric networks: 1 = UND_low; 2 = UND_mid; 3 = UND_high; 4 = UND_extra; 5 = OVHD_low; 6 = OVDH_mid; 7 = OVHD_high; 8 = OVHD_extra-high; 9 = OVHD_ultra-high; 10 = substations.

Figure 1 illustrates, with an interconnected graph, the distributions of electric network types reported in Table 2. Links in the graph show which electric networks are found in the vicinity of the others. As commented above (see Table 2), substations are typically found in the vicinity of underground networks of low and mid voltage and overhead lines of low voltage.

### 3.2. Choice of the Optimal Number of Clusters and Evaluation of Clustering Performance

**Figure 2:**
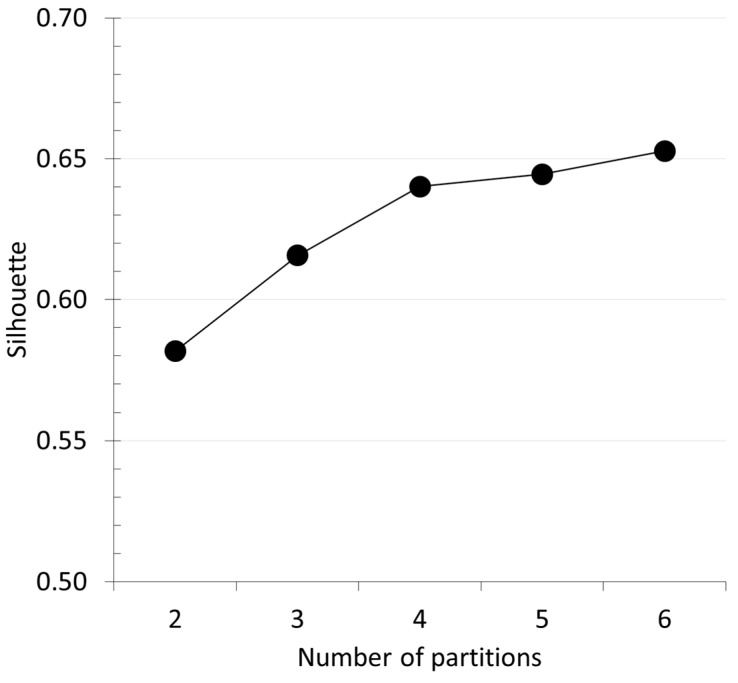
Average silhouette score as a function of the number of partitions.

Figure 2 shows the average silhouette score (see Equation (3)) as a function of the number of clusters used for partitioning the matrix of 1793 × 11 real data. It is possible to see that the score increases as the number of partitions increases from two to six. The increase is steep up to four clusters; at five and six clusters, there is still an increase, but it is minimal if compared to the increase observed from two to four clusters. This means that, using five or six partitions, the quality of the clustering solution would not significantly be better than that obtained with four partitions.

Table 3 shows the number of measurements that the *K*-means algorithm assigned to each cluster (i.e., the cluster size), by varying the number of partitions. As expected, the size of the clusters generally decreased as the number of partitions increased (a deviation from this behavior was seen at cluster #5 in the six-partition solution where the cluster size slightly increased from 264 to 268 if compared to the five-partition solution). In particular, the minimum size of the clusters was seven for the five-partition solution and three for the six-partition solution.

It is a good and consolidated practice to choose the number of partitions so as to maximize the silhouette score and, at the same time, have a reasonable cluster size. For the dataset analyzed in the study, a good compromise between silhouette and cluster size was obtained by partitioning the data with four clusters. Last but not least, it is important to note that the solution with four partitions resulted in a silhouette score of nearly 0.65 which is indicative of good quality of the partitioning.

### 3.3. Results of Clustering Analysis

The results obtained with the four-partition solution can be seen in Table 4 which displays the coordinates (in absolute values) of the centroids of the four clusters. Each centroid is a multi-dimensional vector with 11 components, one for each of the 11 analyzed measurement variables. The number of measurements assigned to each cluster can be found in Table 3, in correspondence to the solution with four partitions.

By definition, in *K*-means clustering, the centroid is obtained as the mean of the data assigned to the cluster. As explained in the Methods Section, each cluster centroid can be regarded as the “representative” of the characteristics of the pool of data assigned to the cluster. The analysis of the coordinates of the centroids is thus useful to understand (i) what are the main characteristics of the clusters, (ii) what are the main differences between the clusters, and (iii) which are the variables that better discriminate the clusters. With regard to points (i) and (ii), from the inspection of Table 4, it is possible to see that cluster #1 pooled together children with the highest exposures (average GM value of B in the cluster: 0.146 μT; Q1: 0.066 μT; Q3: 0.220 μT) and the highest number of extra-high and ultra-high voltage overhead lines close to their home or school (mean OVHD_extra-high: 1 line; mean OVHD_ultra-high: 0.8 lines). Cluster #2 contained children with the highest number of high voltage overhead lines (mean OVHD_high: 1 line) in the vicinity and a mid-to-high exposure (average GM value of B in the cluster: 0.053 μT; Q1: 0.014 μT; Q3: 0.051 μT) (please note that here and in other cases reported below, it may happen that the average would be lower than Q3; this is not an error because quartiles are calculated with respect to the median value of the sample distribution and not the mean). Cluster #3 was for children with a mid-to-low GM B value (average: 0.025 μT; Q1: 0.003 μT; Q3: 0.034 μT) and with the highest number of substations (mean value: 0.9 substations) and underground networks of low and mid voltage (mean UND_low: 11.4 cables; mean UND_mid: 4.6 cables) near home or school; finally, cluster #4 contained children with the lowest exposure (average GM value of B in the cluster: 0.019 μT; Q1: 0.002 μT; Q3: 0.016 μT) and the lowest number of underground cables, overhead lines, and substations near their home or school. Cluster #4 contained all 201 measurements coming from sites that apparently were far from any electric network. For these measurements, the average GM value of B was 0.014 μT (Q1: 0.001 μT; Q3: 0.013 μT) which was well comparable to that obtained from the other measurements assigned to the same cluster #4 (average GM value of B: 0.019 μT; Q1: 0.002 μT; Q3: 0.016 μT). In addition, cluster #4 contained all five samples with B > 0.4 μT (GM). As observed in the other samples assigned to the same cluster, these latter samples corresponded to children with the lowest number of underground cables, substations, and overhead lines of mid to ultra-high voltage in the vicinity. This explains why cluster analysis assigned these latter data to cluster #4; from an overall point of view, the characteristics of these samples were very similar to the other samples of cluster #4. A deeper inspection revealed that these five samples were characterized by a slightly higher number of low voltage overhead lines (Q1: 1 line; Q3: 4 lines) than the other samples in the same cluster (Q1: 0 lines; Q2: 2 lines); this might explain the higher exposure values that characterized these latter measurements.

**Figure 3:**
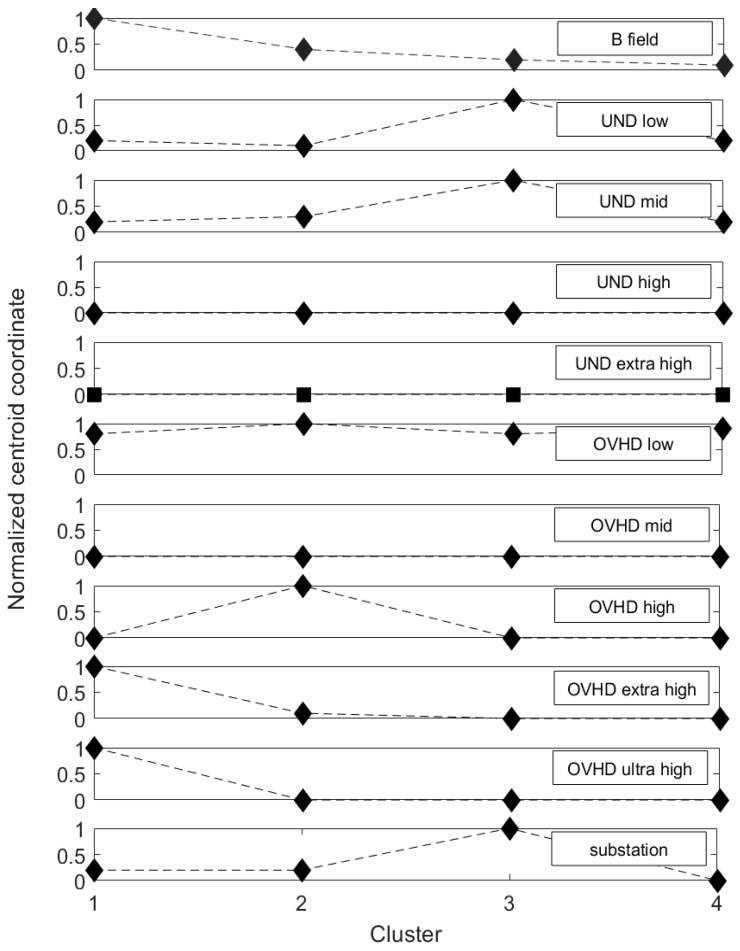
Normalized values of the centroids of the four clusters. Each panel shows the centroid coordinate scaled to the maximum of each of the 11 analyzed variables (as displayed in the panel legends).

With regard to point (iii), to identify which variables better discriminated each exposure cluster, the centroids displayed in Table 4 were normalized by scaling each coordinate separately to the maximum of each column, i.e., to the maximum along each variable. Figure 3 displays the normalized values of the centroid coordinates for each of the 11 variables considered in the study.

From Figure 3, it can be seen that the coordinates of the centroids along the variables UND_high, UND_extra-high, OVHD_low, and OVHD_mid did not vary across the clusters; this means that, on average, data across the different clusters did not show significant differences along these variables. We can thus conclude that these latter variables did not contribute to differentiating the clusters. From a practical point of view, this means that the number of underground networks of high or extra-high voltage and the number of overhead lines of low or mid voltage near home or school were not relevant, alone, to differentiating the characteristics of the four clusters (i.e., the four patterns of exposition).

On the other hand (see Figure 3), all the remaining variables seem to contribute to differentiating the clusters; as a matter of fact, the coordinate of the centroids along these latter variables varied across the clusters, thus meaning that the data assigned to the different clusters have different and unique characteristics. In particular, it is clear that variables OVHD_extra-high and OVHD_ultra-high contributed to differentiating and characterizing cluster #1 (the one with the highest value of B). From Figure 3, it is possible to comment that children living or going to schools near overhead lines of extra-high and ultra-high voltage are most likely characterized by a high value of B. Variable OVHD_high contributed to differentiating cluster #2 (the one with a B field of mid-to-high value); this suggested that children living or going to schools near overhead lines of high voltage are most likely characterized by a mid-to-high B value. Finally, variables substation, UND_low, and UND_mid contributed to differentiating and characterizing cluster #3 (the one with a B field of mid-to-low value); this suggested that children living or going to schools near substations and underground cables of low and mid voltage are most likely characterized by a B field of mid-to-low value.

**Figure 4:**
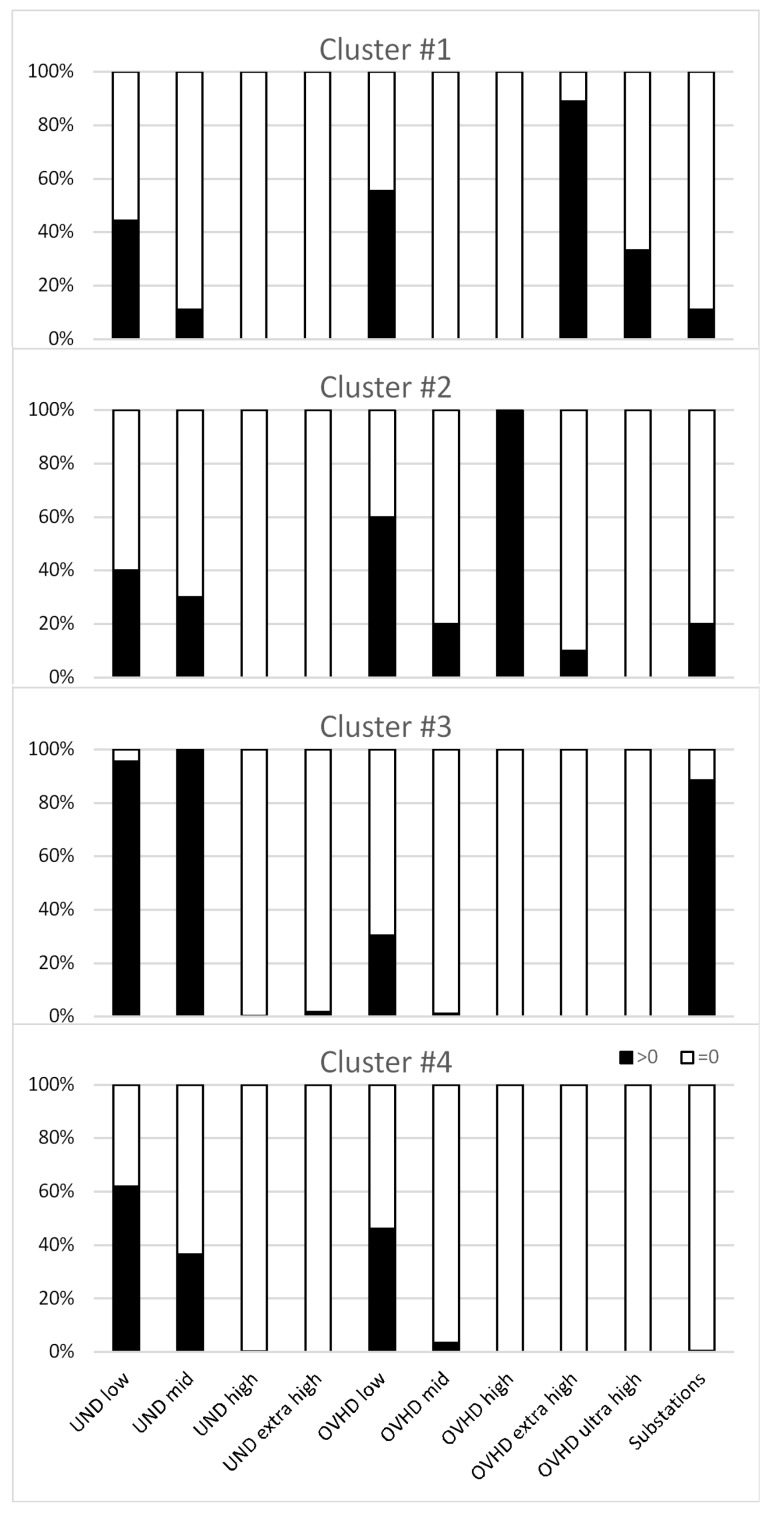
Within cluster analysis. Black bars: Percentage of measurements within each cluster from children living (or going to schools) near underground cables, overhead lines, and substations. White bars: The complement to 100%.

To go deeper in understanding the main features of the four clusters, Figure 4 displays the distribution of indoor measurements within each cluster as a function of the electric network type found near the measurement site.

The first panel of Figure 4 shows that more than 80% of the measurements assigned to cluster #1 were from children near overhead lines of extra-high voltage and that nearly 40% of the measurements were from children near overhead lines of ultra-high voltage. More than 40% of the measurements in cluster #1 were from children near underground low voltage cables and a small percentage (below 10%) were from children close to underground networks of mid voltage or to substations. None of the measurements in cluster #1 were from children near underground networks of high and extra-high voltage or to overhead lines of mid and high voltage.

With regard to cluster #2, 100% of the measurements were from children near at least one overhead line of high voltage; 60% were from children close to overhead lines of low voltage and 40% from children close to underground networks of low voltage. Only a smaller percentage (<30%) of measurements in this cluster were from children who live close to underground cables of mid voltage or overhead lines of mid and extra-high voltage or substations. None of the measurements in cluster #2 were from children near underground networks of high and extra-high voltage or overhead lines of ultra-high voltage. Cluster #3 appears to be mainly characterized by measurements from children living near underground networks of low (97% of the measurements) and mid voltage (100%) and substations (87%). It is important to note that, as displayed in Table 2 and Figure 1, children living near substations are also close to underground cables of low and mid voltage. A very small proportion (1%) of the measurements were from children who live near underground networks of extra-high voltage and overhead lines of mid voltage; none of the measurements in cluster #3 were from children near underground networks of high voltage and overhead lines of high, extra-high, and ultra-high voltage. Finally, cluster #4 was characterized by measurements from children living only near underground cables of low and mid voltage and overhead lines of low voltage.

## 4. Discussion

*K*-means cluster analysis revealed significant and recurrent patterns in personal exposure to ELF MF. The first pattern (cluster #1) corresponded to children living or going to school near overhead lines of extra (225 kV) and ultra-high voltage (400 kV); these children were all characterized by the highest values of MF observed in the dataset (the average GM value of B within the cluster was 0.146 μT). The second pattern (cluster #2) was found for children who live or go to school near overhead lines of high voltage (63/90/150 kV); these children were characterized by a mid-to-high value of MF (the GM value of B averaged within the cluster was 0.0523 μT). The third pattern corresponded to children with the highest number of underground cables of low (400 V) and mid voltage (20 kV) and substations in the vicinity of their home or school; they were characterized by a mid-to-low MF (the average GM value of B within the cluster was 0.025 μT). Finally, the last pattern (Cluster #4) corresponded to children far from power lines and substations; these children were characterized by the lowest values of MF (the average GM value of B within the cluster was 0.019 μT). All these results are in line with [6] (see, in particular, Table 6 in [6] which reports the variables correlated with 24h GM), where the 24 h GM MF exposure was found to be correlated with overhead lines of high to ultra-high voltage, underground cables of low to mid voltage, and substations.

Interestingly, the analysis revealed that underground electric networks of high (63 to 150 kV) and extra-high voltage (225 kV) and overhead lines of low (400 V) and mid voltage (20 kV) were not relevant, alone, to differentiating the characteristics of the four clusters. With regard to underground networks of high and extra-high voltage, the probability of being close to these types of networks is typically low. As expected, because our sample size of 977 children was representative of the French population [6], the resulting dataset contained a small number (12 out of 1793) of measurements made near these types of networks. This would mean that, from a methodological point of view, measurements near underground networks of high and extra-high voltage might have a lower impact on cluster analysis than those made near other types of networks that are more frequently seen in real practice. In parallel, it is also important to note that the contribution of underground networks to total indoor exposure can be small. Although the MF at the centerline of underground cables can be higher than that generated by an equivalent overhead line, the spatial attenuation of the MF is much steeper in these networks than in overhead lines. As a result, the MF to the sides of the cable is usually significantly lower than that of a power line [25,26,27]; for example, the field at 1 m above ground level at a distance of 20 m from the centerline of a 400 kV cable is of the order of 0.10 ÷ 0.20 μT [25,26], whereas the MF of an overhead line of equivalent power is about 2 μT. If measurements are done at more than 1 m above ground level (as in the current study), it is expected that the field will be even lower and of less contribution to total exposure.

A significant percentage of the measurements in the dataset were from children (43.8%) living near overhead lines of low voltage which were found to be of low efficacy in differentiating the clusters. A possible explanation for this aspect could be that these networks marginally contributed to the measured exposure. This is also in line with [6], where no correlation was found between the 24 h GM MF exposure and low voltage lines. Finally, with regard to mid voltage overhead lines, they were found to have a marginal contribution in differentiating the clusters; again, one possible explanation for this result could be the small number of children in the dataset that live near mid voltage overhead lines (58 out of 1793). In addition, as commented in [6], mid voltage lines were mainly found in rural areas characterized by lower exposure values if compared to the exposure measured in highly urbanized areas, such as in Paris. This last evidence would mean that, if present, mid voltage lines marginally contribute to the exposure.

As described above, the pattern observed in cluster #3 concerned children who live or go to school near substations and underground networks of low and mid voltage. As commented above, the MF of underground networks could be very low when measured at a distance of 20 m and even lower at greater distances. Our dataset contained information regarding the presence of low and mid voltage cables within 40 m from a child’s home or school; thus it could be that the contribution of low and mid voltage cables to our exposure data might be very low. On the other hand, according to the network distribution observed in our dataset, children who live near substations usually have underground networks of low and mid voltage in close proximity. Thus, we can conclude that the true differentiating variable that characterized mainly cluster #3 was the presence of substations near home or school, whereas the presence of underground networks of low and mid voltage was only a consequence of the configuration of power grids in the analyzed dataset.

The scarce contribution of underground networks to MF at distances greater than 20 m and the results from cluster analysis seem to suggest that, in future personal exposure campaigns, it would be interesting to record the presence of underground networks at distances below 20 m (i.e., at a distance where their MF is not marginal). This would provide additional information needed to better determine the role of these networks in differentiating the exposure patterns.

With regard to the uncertainty due to measurements of the B field near the sensitivity threshold of the personal dosimeter, it could be that the GM calculated from the values of B measured with the device was below the sensitivity threshold (equal to 0.001 μT). However, we can assume that the impact of this aspect should be marginal because clusters were identified by the *K*-means algorithm by considering not only the value of B but also all the other 10 variables related to the number of electric networks in the vicinity of the measurement position.

It is important to note that the geolocalization of electric networks might have some slight uncertainties. As a matter of fact, the geolocalization of electric networks was done from the physical address of a child’s home or school; that is from the mailbox, which is a point closer to the street. This means that the exact and real position of the child with respect to the electric network nearby cannot be known without uncertainties, especially in those locations where the child can move around large areas, such as in schools. It seems reasonable to assume that the impact of this uncertainty should be marginal. At the moment, it is not possible to give an exact and quantitative estimation of this aspect using the data analyzed in the present study. It could be interesting in further studies to go deeper in analyzing the impact of this uncertainty, for example by considering both the approximate locations (derived from the physical address of the child’s home or school) and, if available, the exact GPS position of the child.

Last but not least, we are planning to extend this cluster analysis by also considering as additional variables indoor sources of the exposure, such as the type of heating in the child’s home or school, to discover how they might impact on the exposure scenario.

## 5. Conclusions

Cluster analysis was applied on personal exposure measurements recorded 24 h for one day from children living in France. The analysis revealed four clusters of indoor ELF MF exposure characterized by significantly different patterns of underground networks, power lines, and substations near a child’s home or school. Among the electric networks considered in the study, the analysis also revealed that the presence of overhead lines of high to ultra-high voltage and substations near home or school greatly contributed to differentiating the four clusters of indoor MF exposure. The present analysis seems to indicate that the presence of underground networks and low to mid voltage overhead lines within 20 ÷ 40 m from home or school did not contribute to cluster differentiation (probably because of the spatial attenuation of the B field which is not negligible at such distances). Although the present analysis revealed that children living within 20 ÷ 40 m from these latter networks were typically characterized by the lowest exposures, it might be interesting, in future measurement campaigns, to collect more samples at distances lower than 20 ÷ 40 m, i.e., at distances where the B field generated by these networks would only be partially attenuated. This would give us the chance to assess if the lowest exposures evidenced in the present study were due to the type of network, irrespective of the distance, or if they were due to a combined effect of the type of network and the distance from the measurement location.

## Figures and Tables

**Table 1:** Distribution of electric networks in the analyzed dataset. The table shows the number of indoor measurements close to each network type. The last two columns on the right show, for each network type, the maximum and mean number of power lines and substations near the measurement site (i.e., home or school).

Network Type	Number of Indoor Measurements (% of All Indoor Measurements) ^1^	Number of Power Cables, Power Lines and Substations	
		Max	Mean
UND_low	1198 (66.8%)	59	3.9
UND_mid	820 (45.7%)	27	1.3
UND_high	5 (0.3%)	2	0.0
UND_extra-high	7 (0.4%)	2	0.0
OVHD_low	786 (43.8%)	16	1.1
OVHD_mid	58 (3.2%)	5	0.0
OVHD_high	10 (0.6%)	1	0.0
OVHD_extra-high	9 (0.5%)	2	0.0
OVDH_ultra-high	4 (0.2%)	3	0.0
Substation	246 (13.7%)	2	0.1

^1^ Number of all indoor measurements = 1793.

**Table 2:** Distribution of electric networks in the analyzed dataset (cont.d). The main diagonal (shaded background) shows the number (and %) of measurements from children whose home or school is in proximity of only a single type of network, whereas data below the main diagonal show the number (and %) of measurements in close proximity to two different types of networks at the same time.

Network Type		UND				OVHD					Substations
		Low	Mid	High	Extra-High	Low	Mid	High	Extra-High	Ultra-High	
UND	low	317 (17.7) ^1^									
	mid	696 (38.8)	23 (1.3)								
	high	4 (0.2)	5 (0.3)	0							
	extra-high	7 (0.4)	6 (0.3)	0	0						
OVHD	low	431 (24.0)	352 (19.6)	4 (0.2)	1 (0.1)	228 (12.7)					
	mid	22 (1.2)	11 (0.6)	0	0	41 (2.3)	4 (0.2)				
	high	4 (0.2)	3 (0.2)	0	0	6 (0.3)	2 (0.1)	1 (0.1)			
	extra-high	5 (0.3)	2 (0.1)	0	0	5 (0.3)	0	1 (0.1)	0		
	ultra-high	2 (0.1)	1 (0.1)	0	0	1 (0.1)	0	0	2 (0.1)	0	
	Substation	228 (12.7)	237 (13.2)	1 (0.1)	1 (0.1)	87 (4.9)	11 (0.6)	2 (0.1)	2 (0.1)	0	0

**^1^** Number of all measurements = 1793.

**Table 3:** Cluster size (i.e., number of measurements) for different partitioning solutions, from 2- to 6-cluster solutions.

Cluster #	Number of Partitions				
	2	3	4	5	6
1	269	10	9	7	3
2	1524	267	10	9	5
3		1516	267	10	7
4			1507	264	10
5				1503	268
6					1500

**Table 4:** Coordinates of the centroids of the four clusters for each of the 11 analyzed measurement variables (from “B” to “Substation”).

Cluster #	B (μT)	UND (N)				OVHD (N)					Substation (*N*)
		Low	Mid	High	Extra-High	Low	Mid	High	Extra-High	Ultra-High	
1	0.146	2.4	0.9	0.0	0.0	1.0	0.0	0.0	1.0	0.8	0.2
2	0.053	1.2	1.3	0.0	0.0	1.3	0.2	1.0	0.1	0.0	0.2
3	0.025	11.4	4.6	0.0	0.0	1.0	0.0	0.0	0.0	0.0	0.9
4	0.019	2.6	0.7	0.0	0.0	1.2	0.0	0.0	0.0	0.0	0.0
